# Physicochemical and Rheological Properties of Floury Rice Powder with Different Particle Sizes: Effects on Gluten-Free Sponge Cake Qualities

**DOI:** 10.3390/gels11100789

**Published:** 2025-10-01

**Authors:** Hyebin Jeon, Jungae Lee, Tae Gyu Nam, Hyunwook Choi, Hyun-Seok Kim

**Affiliations:** 1 Department of Food Science and Biotechnology, Graduate School, Kyung Hee University, Yongin 17104, Republic of Korea; hyebin_0126@khu.ac.kr; 2 Department of Food Science and Biotechnology, Graduate School, Kyonggi University, Suwon 16227, Republic of Korea; lje0099@naver.com (J.L.); tgzoo0706@kyonggi.ac.kr (T.G.N.); 3 Department of Food and Nutrition, Jeonju University, Jeonju 55069, Republic of Korea; 4 Department of Food Science and Biotechnology, Institute of Life Science and Resources, Kyung Hee University, Yongin 17104, Republic of Korea

**Keywords:** floury rice powder, butter sponge cake, gluten-free, particle size, viscoelasticity, gelling tendency

## Abstract

This study investigated the physicochemical and rheological properties of floury rice powder (FRP) with different particle sizes and their effects on the quality characteristics of gluten-free butter sponge cake. Soft rice grain (Baromi2 variety) was dry-milled and sieved into four fractions: FR1 (60 mesh overs), FR2 (60–80 mesh), FR3 (80–100 mesh), and FR4 (100 mesh throughs). FRP fractions were analyzed for chemical composition, swelling power, solubility, gelatinization, pasting viscosity, and viscoelastic property. Gluten-free cakes made using a whole-egg foam method were evaluated for morphological structure, baking loss, moisture, specific volume and firmness. With decreasing FRP particle size, there were increasing trends in solubility, pasting viscosity, resistance to deformation, viscoelastic attributes (G′ and G″), and gel rigidity. FR3 and FR4 cakes exhibited flat and puffy loaves compared to FR1 and FR2 cakes with loaf collapses. The finer FRP enhanced the morphological balances of the cakes. Increasing trends in specific volume and firmness were observed as FRP particle size decreased. These results paralleled the solubility, pasting, rheological, and gelling properties of FRP itself. Overall, the results suggest that the rheological and gelling properties of FRP may play a role in determining the quality of gluten-free sponge cakes. In addition, FRP with a particle size of 80–100 mesh appears most appropriate for gluten-free sponge cake.

## 1. Introduction

Gluten in wheat flour is a complex protein network that develops through physical and chemical interactions between gliadin (prolamins) and glutenin [1]. The quality of gluten varies depending on the variety, genotype, and cultivation conditions of wheat, as well as the composition ratio and molecular size of gliadin and glutenin, and the processing method of wheat flour [1,2,3]. These factors play crucial roles in determining the development and viscoelasticity of dough, and the processing suitability and end-use quality of wheat flour-based products such as breads, cakes, cookies, pastas, noodles, and other processed foods containing wheat flour as an additive [1,2,3]. However, the consumption of gluten and gliadin can cause celiac disease, a genetic disorder [1]. The prevalence of celiac disease is reported to be 1–2% of the global population, but this number is gradually increasing with advancements in diagnostic technology [1,2]. Including the uncounted gluten-sensitive individuals, it is expected that many people worldwide suffer from the consumption of wheat-based food products [2,4]. Celiac disease is a chronic inflammatory intestinal disease caused by an autoimmune response to prolamins, particularly gliadin [1,2,4]. This disease can lead to nutritional deficiencies and imbalances, often accompanied by diarrhea, anemia, weight loss, and motor dysfunction, and in severe cases, death [2,4]. Celiac disease, which cannot be cured by surgical treatment, can be alleviated solely by eliminating gluten and gliadin from the diet [1,2,4]. This is achieved by excluding foods prepared with food sources and additives made from *Triticale* sp. crops (wheat, rye, barley, and triticale) [1,2,4]. Therefore, there is growing interest worldwide in the development and consumption of gluten-free food products [1,2]. The global market for gluten-free foods is anticipated to grow from 6.7 billion USD in 2022 to 14 billion USD by 2032 [2].

Aside from replacing raw and processed materials from rye, barley, and triticale, gluten-free foods can be achieved by substituting wheat flour, widely used in the food industry, with other flours that do not contain either gluten or gliadin [1,2]. Currently, rice flour is gaining attention as a raw material to fully or partially replace wheat flour in gluten-free foods due to its reproducibility, mild flavor, high whiteness, easy digestibility, hypoallergenic properties, and global consumption [2]. However, rice flour cannot replicate the physical and rheological functionalities of gluten in wheat flour due to minimal or no gliadin [3]. This limits the use of rice flour as an alternative to wheat flour in traditional bakery and noodle products. Consequently, many studies have been conducted to enhance the quality, sensory, and nutritional attributes of gluten-free bakery [5,6,7,8,9,10,11,12,13,14,15,16,17,18,19,20,21,22,23,24,25,26,27,28,29] and noodle [30,31,32] products in the absence of gluten. These studies focus on the impacts of the rice cultivar [7,12,17,18], milling method of rice grain [5,10,13,31], particle size of rice flour [5,6,7,8,9,10,13,16,30], thermo-mechanical treatment of rice flour [18,19], and the addition of additives such as hydrocolloids [3,22,23,24], proteins [14], and other crop flours [21,22,23,24,25,26,27,28,29,30] to rice flour-based formulations. Among these factors, the particle size of rice flour has shown relatively consistent effects on the quality attributes of gluten-free rice breads [6,7,8,9,10] and cakes [13,16]. These studies [6,7,8,9,10,13,16] commonly demonstrated that the pasting viscosity of rice flour, along with the viscosity and viscoelastic attributes—storage modulus (elastic character, G′) and loss modulus (viscous character, G″)—of the batter, increased with decreasing particle size. This resulted in small, evenly distributed air cells in crumb, as well as higher specific volume and lower firmness in gluten-free breads and cakes. However, the viscosity and viscoelastic properties of the batter were not always directly associated with the quality attributes of gluten-free breads and cakes. This may be due to slippage between the smooth spindle or geometry and the oily batter, which can lead to underestimation or distortion of the rheological properties of the batter. Moreover, gluten-free rice layer cakes made with thoroughly stirred batter showed a relatively consistent relationship between the quality attributes of the cake and the rheological attributes of the batter [13,16]. In contrast, this relationship was rare in gluten-free rice sponge cakes made with the batter that is gently folded to minimize the collapse of egg foam [13,16].

Therefore, the objective of this study was to investigate the physicochemical, pasting, and viscoelastic properties of floury rice powder (FRP) with different particle sizes, to evaluate the quality characteristics of gluten-free butter sponge cakes made with FRP, and to verify whether these quality characteristics could be associated with and explained by the FRP properties. The results obtained in this study could provide insights into how flour sources affect the quality attributes of the gluten-free cakes and offer criteria for selecting flour sources for these cakes. In addition, the FRP used in this study was dry-milled from the soft, short grains of the Baromi2 variety, developed by breeding Suweon542 and Jopyeong rice, which belong to the Japonica variety, to replace wheat flour [33,34]. The morphological characteristics of rice starch in the FRP are very similar to those of wheat starch rather than common rice starch [34]. Thus, this study explored the potential of FRP as a main ingredient for gluten-free bakery products, based on these results.

## 2. Results and Discussion

### 2.1. Characteristics of Floury Rice Powder (FRP) Fractionated by Particle Size

#### 2.1.1. Chemical Composition

The proximate compositions and total starch contents of FRP fractions and soft wheat flour are shown in Table 1. The crude protein, crude fat, carbohydrate, and total starch contents of FRP fractions ranged from 5.8% to 8.1%, 1.6% to 2.2%, 89.2% to 92.1%, and 73.4% to 77.6%, respectively. The crude ash content remained constant at 0.5%, regardless of FRP particle size. These results are consistent with those reported by the previous research [34] that compared the physicochemical properties of flour-type rice (Baromi2 variety) with common rice (Samkwang variety). Notably, the higher crude fat content (1.6–2.2%) of FRP fractions compared to common rice flours (0.3–0.5%) [5,11,34] is attributed to the inherent characteristic of Baromi2 floury rice [34,35]. Meanwhile, the crude protein and crude lipid contents of FRP fractions gradually decreased with decreasing particle size, while carbohydrate and total starch contents increased. These patterns are consistent with reports from previous studies [6,7,8,13,35] and are attributed to a relative increase in rice starch content within FRP as the particle size decreases [5,6,34]. FRP exhibits irregular granular structures, in which rice starch granules are tightly incorporated into the rice protein body [34]. The smallest rice starch granules (1–5 μm in diameter) [36] are prevalently released from the rice protein body through mechanical grinding. These individual rice starch granules can more easily pass through the sieve with smaller pore sizes. Consequently, the FRP fraction with a smaller particle size distribution may contain a relatively higher content of rice starch, along with increased carbohydrate content. In addition, the total dietary fiber content of FRF fractions ranged from 1.7% to 1.9%, with no significant difference (Table 1).

Meanwhile, the crude protein, crude lipid, crude ash, carbohydrate, total starch, and total dietary fiber contents of soft wheat flour were 7.3%, 0.9%, 0.3%, 91.5%, 71.6%, and 2.1%, respectively (Table 1). The crude protein content of soft wheat flour was similar to those of FR2 and FR3 in FRP, while the carbohydrate content was between FR3 and FR4. Crude lipid, crude ash, and total starch contents were lower than those of the FRP fractions. The total dietary fiber content of soft wheat flour did not significantly differ from that of the FRP fractions.

#### 2.1.2. Swelling Power and Solubility

Swelling power and solubility of FRP fractions and soft wheat flour are presented in Table 2. The swelling power of the FRP fractions ranged from 22.2 g/g to 22.5 g/g with no significant differences and was higher than that of soft wheat flour. This result is consistent with the findings reported by previous studies [6,9,30] that demonstrated no or little impact of particle size on the swelling power of rice flour at high temperature (80–100 °C), although the swelling power was influenced by milling methods. In addition, the swelling power of rice flour contributes to the increased consistency of batter in gluten-free bakery products [9]. This increase in batter consistency (or viscosity) reduces the specific volume of gluten-free bread [10] and layer cake [14]. Therefore, in this study, it seems unnecessary to consider changes in batter consistency by FRP fractions on the quality characteristics of gluten-free butter sponge cakes. For solubility, FRP fractions significantly increased from 12.3% to 15.1% with decreasing particle size, consistent with the results of previous studies [6,9,30]. This result may be attributed to the solubilization of rice starch and non-starch carbohydrates in FRP, following swelling and gelatinization, due to the poor water solubility of rice protein [37]. This is further supported by the increased carbohydrate and total starch contents of FRP fractions with decreasing particle size (Table 1). Meanwhile, soft wheat flour (20.0%) exhibited higher solubility than FRP fractions (12.3–15.1%), even though its carbohydrate and total starch content were lower than those of FR4 and FR1, respectively (Table 1). This may be attributed to the presence of water-soluble pentosans (0.5–0.8% in wheat flour) [38] and the higher solubility of wheat starch B-type granules (less than 10 μm in diameter) [39].

#### 2.1.3. Thermal Property

Gelatinization temperatures and enthalpies of FRP fractions and soft wheat flour are shown in Table 3. The gelatinization onset temperature of FRP fractions decreased from 57.7 °C to 55.4 °C with decreasing particle size. However, the gelatinization peak (67.4–68.0 °C) and completion (73.8–74.7 °C) temperatures, as well as the gelatinization enthalpies (7.2–9.1 J/g) were not significantly different between FRP fractions. These results are consistent with the report by Qin et al. (2021) [6] but partially agree with the result by Park et al. (2014) [8], which reported no significant differences in gelatinization onset temperatures between rice flours with different particle sizes. This inconsistency may be due to differences in milling methods. The former study [6] prepared rice flour samples (closer to the rice powder in this study) by blending flours subjected to dry and semidry milling, whereas the latter study [8] used rice flour prepared through semidry milling. Overall, the gelatinization properties of rice flour appear to be less influenced by particle size due to subtle deviations in gelatinization temperatures and enthalpies among FRP fractions. Meanwhile, soft wheat flour exhibited a higher gelatinization onset temperature (57.9 °C vs. 55.4–55.7 °C), but lower gelatinization peak (64.6 °C vs. 67.4–68.0 °C) and completion (70.7 °C vs. 73.8–74.7 °C) temperatures, and lower gelatinization enthalpy (3.2 J/g vs. 7.2–9.1 J/g) compared to FRP fractions. This result indicates that soft wheat flour easily gelatinizes at relatively low temperatures with less energy than FRP. This suggests that rapid gelatinization of soft wheat flour contributes more to the formation of small, homogeneous air cells and stable volume expansion in cakes [13,14]. For retrogradation after 1 week storage at 4 °C, melting enthalpies of FRP fractions ranged from 1.2 J/g to 1.4 J/g with no significant difference, and were higher compared to soft wheat flour (0.5 J/g). This may imply that gluten-free rice cakes exhibit a firmer texture than wheat flour-based cakes.

#### 2.1.4. Pasting Property

**Figure 1:**
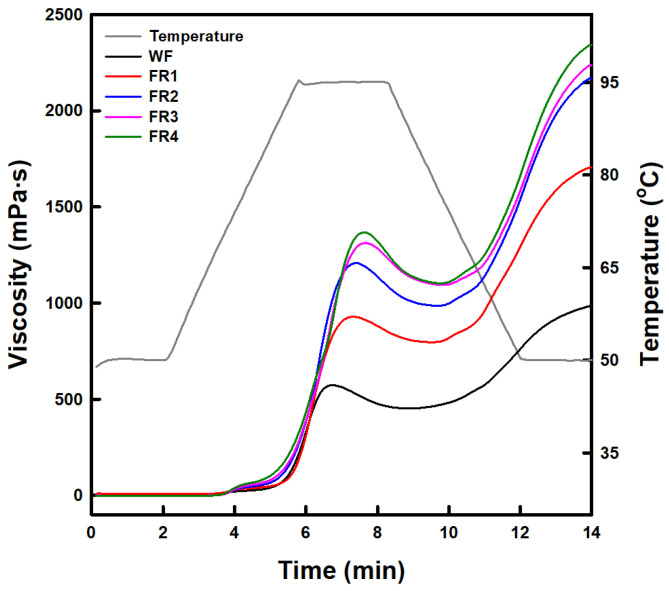
Pasting viscosity profiles of soft wheat flour (WF) and floury rice powder (FR1–FR4) fractionated by particle size distribution.

Pasting viscosity profiles and parameters (determined at a total solid content of 7.5%) of FRP fractions and soft wheat flour are presented in Figure 1 and Table S1, respectively. The overall pasting viscosity profiles of FRP fractions increased with decreasing particle size (Figure 1), resulting in significant increases in peak, trough, and final viscosities (Table S1). Increasing trends in breakdown and setback viscosities (calculated from peak, trough, and final viscosities) were also observed with decreasing particle size. However, there was no significant difference in breakdown viscosity between F2 and F3 and the final viscosity of F2 was significantly higher than that of F3 (Table S1). These results are consistent with the findings of Qin et al. (2021) [6], but not with other previous studies [8,9,13] that did not find a direct impact of particle size on the pasting viscosities of rice flour. This inconsistency may be due to differences in rice variety, milling method, milling machine, and fractionation method used for preparing and fractionating rice flour. Meanwhile, the peak, final, and setback viscosities are crucial for forming the gel-like network structure of rice flour paste in gluten-free rice cakes [13]. In this study, the increase in these pasting viscosities of FRP fractions with decreasing particle size may be attributed to the relative increase in rice starch content, following a reduction in rice protein content [6]. Rice protein rarely functions as a thickener and gelling agent due to its poor solubility in water [37]. Instead, rice protein can absorb water [37], restricting the water availability of the starch in rice flour. This reduces and delays the hydration, swelling, and gelatinization of rice starch, preventing viscosity development and gel-like structure formation [40]. Consequently, rice flour with low rice protein content (FR4 in this study) appears suitable for forming and stabilizing the network structure of rice flour-based gluten-free bakery products. For soft wheat flour, the pasting viscosity was lower across all points of the programmed temperature profile compared to FRP fractions. This result may be due to the presence of wheat protein, pentosans, and wheat starch B-type granules, which prevent viscosity development [38,39,40], as well as the relatively low total starch content.

#### 2.1.5. Strain Sweep Characteristic

**Figure 2:**
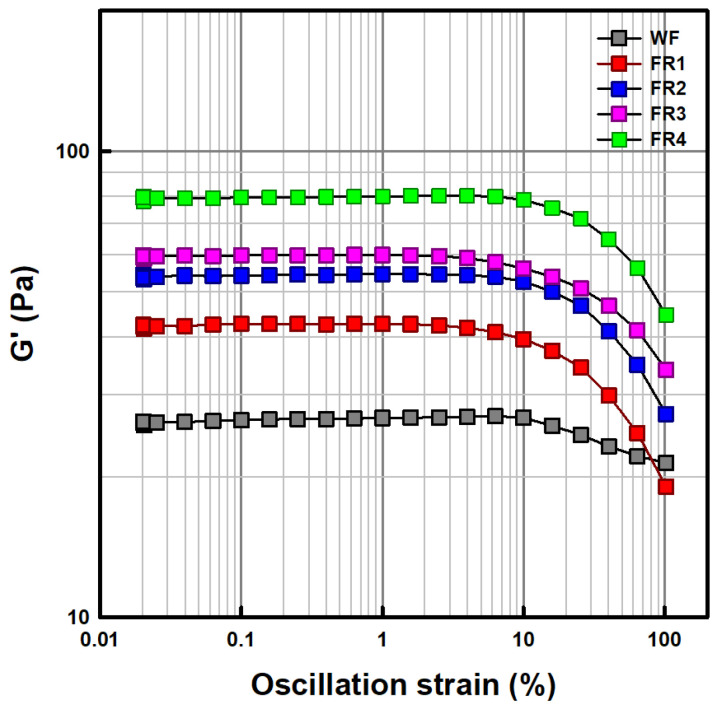
Strain sweep profiles of soft wheat flour (WF) and floury rice powder (FR1–FR4) fractionated by particle size distribution.

Strain sweep profiles of gels (prepared at a total solid content of 7.5%) from FRP fractions and soft wheat flour are shown in Figure 2. The storage moduli (G′) of all samples were higher than the loss moduli (G″) over the oscillation strain range of 0.01–100% (Figure S2), indicating solid-like behavior [41]. The upper limit of the linear viscoelastic region (LVR), where the G′ value remained at a plateau, was found to be 2.6% strain for FR1, 4.0% strain for FR2 and FR3, 6.5% strain for FR4, and 10% strain for soft wheat flour. This result indicates that the LVR of FRP fraction gels extends with decreasing particle size. Based on this, 1% strain was chosen for frequency and temperature sweep tests. Additionally, the altitude in G′ values of the FRP fractions and soft wheat flour within the LVR coincided with the peak and final viscosities obtained from the RVA test (Figure 1). Two distinct parameters, critical strain (γ_c_) and flow strain (γ_f_), were derived from strain sweep profiles [41,42]. The γ_c_ value is commonly determined as the strain value at the intersection of the linear equations obtained by extrapolating the respective LVR and non-linear regions of the strain sweep profile in the G′ value, indicating the strain at which the gel structure begins to break down under continued oscillation strain. The γ_f_ value is determined as the strain at which G′ and G″ become equal (G′ = G″), indicating the strain at which the gel state transitions into the liquid state by continued oscillation strain. In this study, the γ_f_ values of FRP fractions were unable to be determined because crossover points where G′ and G″ profiles intersect were not observed over the oscillation strain range. Meanwhile, the γ_c_ values increased in the order of FR4 (29%) > FR3 (23.5%) > FR2 (22%) > FR1 (17%) > soft wheat flour (10%). This result may suggest that the finer the particle size of FRP, the greater its resistance to deformation by air cell expansion during the baking of butter sponge cake batters.

#### 2.1.6. Frequency Sweep Characteristic

Previous studies [6,11,15,18,20,30] investigated the rheological properties of batters in gluten-free breads and cakes, whereas this study focused on the rheological properties of FRP and soft wheat flour. This is because the butter sponge cake batter was prepared by adding FPR and soft wheat flour after the full formation of the whole egg foam. This approach may lead to the continuous collapse of the whole egg foam, the fundamental structure of the sponge cake batter, during rheological testing due to continued oscillation, making it difficult to obtain consistent rheological data. Moreover, since FRP or soft wheat flour exists on the surfaces of the whole egg foam and at the interfaces between air cells in the foam, where they undergo swelling, gelatinization, and gelation during baking, they reinforce the rigidity and integrity of the whole egg foam and suppress air leakage from the batter. Therefore, it is crucial to investigate the rheological properties of the flour itself in this study. The dynamic viscoelastic characteristics of gels (prepared at a total solid content of 7.5%) from FRP fractions and soft wheat flour were investigated as a function of angular frequency. The frequency sweep plots and parameters (at 1 Hz) of gels from FRP fractions and soft wheat flour are shown in Figure S3 and Table 4, respectively. The G′ values of all samples were higher than the G″ values over the tested frequency range (Figure S3), and the tan δ values were lower than 1 at all frequency points, indicating gel-like or solid-like behavior [11,30]. The viscoelastic moduli (G′ and G″) of all samples were slightly dependent on frequency without a crossover between G′ and G″, suggesting that the gel-to-sol transition was absent in these systems [42] and that the gels exhibit a relatively weak elastic structure [11]. Regarding viscoelastic parameters at 1 Hz (Table 4), the G′, and G″ values of FRP fractions increased with decreasing particle size, although no significant differences were observed. These results are consistent with the peak and final viscosities in the RVA testing and the G′ values in the strain sweep test. The tan δ values remained constant at 0.2, suggesting that the viscoelastic character of the FRP gels is not influenced by particle size. In addition, the complex viscosity (η*) values, a measure of overall viscous and elastic behavior, followed the patterns observed in the G′ and G″. This result further supports that the gel from the smaller FRP has greater resistance to deformation or flow. The η* values of a given sample decreased with increasing frequency, indicating shear-thinning behavior. Meanwhile, the viscoelastic behavior and characteristics of soft wheat flour were very similar to those of FR2 (Table 4).

#### 2.1.7. Temperature Sweep Characteristic

**Figure 3:**
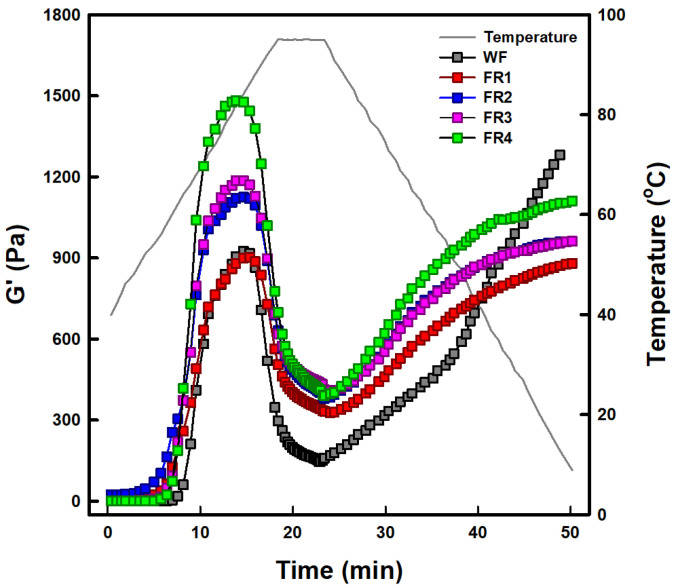
Temperature sweep profiles of soft wheat flour (WF) and floury rice powder (FR1–FR4) fractionated by particle size distribution.

Changes in viscoelastic parameters of FRP fractions and soft wheat flour were investigated using a temperature sweep test at a total solid content of 15%. The temperature sweep plots of all samples are shown in Figure 3. The G′ values of all samples were much higher than the G″ values across all points of the predetermined temperature profile (Figure S4), indicating that all samples exhibit viscoelastic solid-like behavior, as observed in strain (Figure S2) and frequency (Figure S3) sweep tests. The patterns in temperature sweep profiles of FRP fractions were similar to those in pasting viscosity profiles (Figure 1). Peaks, indicating maximum swelling, of FRP fractions were observed at 82–86 °C. The G′ values at these peaks increased in the order of FR4 (1482.9 Pa) > FR3 (1186.7 Pa) > FR2 (1124.7 Pa) > FR 1 (902.9 Pa). The G′ values of the final gels at 5 °C increased in the order of FR4 (1111.0 Pa) > FR3 (962.6 Pa) > FR2 (960.7 Pa) > FR 1 (880.4 Pa). These results are attributed to relatively higher starch and lower protein contents resulting from particle size reduction (Table 1). The presence of rice protein in FRP suppresses the hydration, swelling, and gelatinization of rice starch granules which are individual and incorporated into the rice protein body. This also inhibits the leaching of starch molecules from FRP granules [37,40], ultimately weakening the gel rigidity—its ability to resist deformation and maintain structure. Meanwhile, the temperature sweep plot of soft wheat flour differed from its pasting viscosity profile (Figure 1), which was lower across all points of the predetermined temperature profile than that of the FRP fractions. However, in the temperature sweep plot, soft wheat flour showed an increasing trend in G′ values similar to that in FR1 and reached a peak G′ value (925.1 Pa) exceeding that of FR1 (902.9 Pa). During the cooling stage from 95 °C to 5 °C, the G′ values dramatically increased and eventually exceeded the G′ value of FR4 (1111.0 Pa). The final G′ value of the gel from soft wheat flour was 1281.2 Pa. Considering that soft wheat flour exhibited similar crude protein content to that of FR1-FR3 and lower total starch content than FRP fractions (Table 1), these results may be due to gluten development caused by the continuous oscillation of a relatively higher concentration of soft wheat flour placed in the narrow space between a Peltier plate and geometry during the temperature sweep test.

#### 2.1.8. Thermo-Mechanical Properties

**Figure 4:**
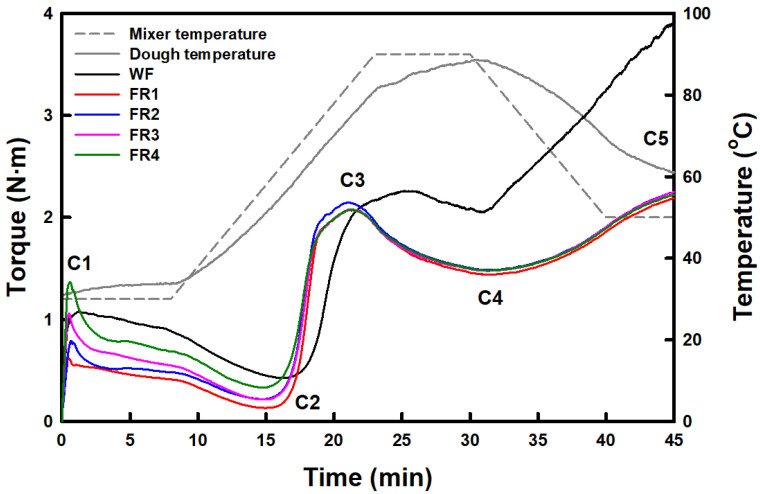
Mixolab profiles of soft wheat flour and floury rice powder (FRP) fractionated by particle size distribution.

Soft wheat flour developed noticeable G′ values across the predetermined temperature profile (Figure 3), different from its pasting viscosity profile. This result was explained in Section 2.1.7 by gluten development during the temperature sweep test. Another possibility could be the differences in total solid content applied between the temperature sweep (15%) and RVA (7.5%) tests. Thus, changes in the consistency (measured by torque) of FRP fractions and soft wheat flour during mixing, followed by heating, were investigated using Mixolab in a high concentration regime (batter or dough). The Mixolab profiles and parameters are shown in Figure 4 and Table S2, respectively. In the Mixolab profile, C1 and C2 indicate the maximum torque of batter or dough at the initial mixing stage and the minimum torque derived from protein network weakening, respectively [31]. C3 represents the maximum torque resulting from starch gelatinization, C4 indicates the hot gel stability, and C5 corresponds to the degree of starch retrogradation or gelation at the cooling stage [31]. The C1 and C2 values of FRP fractions increased with decreasing particle size. The differences between C1 and C2 values in FRP fractions also increased with decreasing particle size, indicating that finer FPR granules result in weaker resistance of their batter against mixing [32]. The C1 value of soft wheat flour was similar to that of FR3, and the C2 value was significantly higher than that of the FRP fractions. Although the difference between C1 and C2 values in soft wheat flour was similar to that in FR2, the torque profile gradually decreased compared to that of FRP fractions. This result may be attributed to the gluten development in soft wheat flour during the mixing stage [43]. The C3, C4, and C5 values of FRP fractions ranged from 2.08 to 2.17 N·m, 1.44 to 1.50 N·m, and 2.31 to 2.38 N·m, respectively, with significant differences (Table S2). However, the variations in C3, C4, and C5 values among FRP fractions are negligible, suggesting that the gelatinization and gelling properties of FRP batters may rarely be influenced by particle size. In soft wheat flour, the C3, C4, and C5 values were significantly higher than those of the FRP fractions. This result indicates that the rheological behavior of soft wheat flour during mixing and heating becomes more pronounced in the high concentration regime (batter or dough) because gluten development is facilitated.

### 2.2. Characteristics of Butter Sponge Cake: Effects of FRP with Different Particle Size

#### 2.2.1. Morphological Characteristics

**Figure 5:**
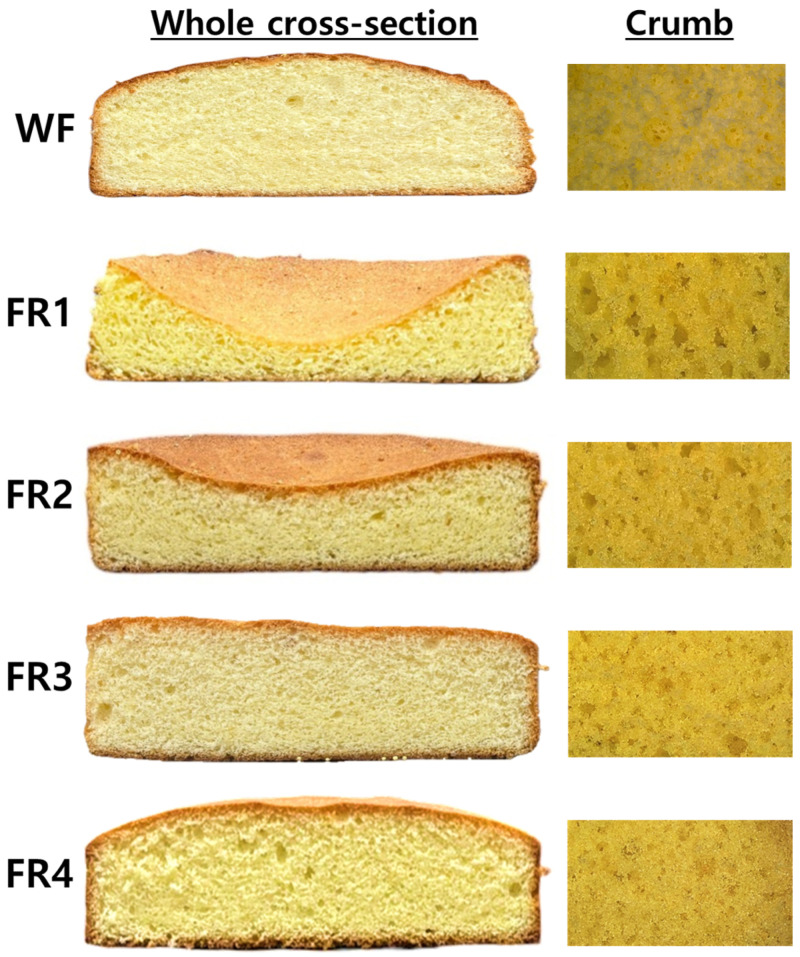
Whole cross-section and crumb images of butter sponge cakes prepared using soft wheat flour (WF) and floury rice powder (FR1–FR4) fractionated by particle size distribution.

Whole cross-section and crumb images of butter sponge cakes prepared using FRP fractions and soft wheat flour are shown in Figure 5. The specific gravity of the sponge cake batters remained similar to exclude the influence of the amount of air incorporated into the batter on the characteristics of the sponge cakes. The soft wheat flour (WF) sponge cake exhibited the puffy loaf structure, with small air cells evenly distributed throughout the crumb. In FRP-based sponge cakes, loaf collapse was observed in the FR1 and FR2 sponge cakes, with the collapse being more pronounced in FR1, which had the largest particle size. The FR3 sponge cake exhibited a flat loaf structure, while the FR4 sponge cake had a puffy loaf structure similar to that of the WF sponge cake. The size of air cells in the crumb became increasingly smaller from FR1 to FR4 sponge cakes, corresponding to the use of FRP with decreasing particle size. The crumb structure in the FR4 sponge cake was closer to that in the WF sponge cake.

The volume, symmetry, and uniformity indexes of butter sponge cakes prepared using FRP fractions and WF are presented in Table 5. The volume index, a measure of cake volume, significantly increased in the order of FR3 (126.9 mm) > FR4 (116.7 mm) > FR2 (88.8 mm) > FR1 (64.7 mm). However, sponge cakes made with FRP fractions exhibited much lower volume indexes than the WF sponge cake (185.4 mm). The symmetry index, indicating how evenly the height of the cake is distributed from its center to its edge [16,44], was −8.1 mm and −10.0 mm for the FR1 and FR2 sponge cakes with loaf collapse, showing no significant differences. The FR3 sponge cake with a flat loaf had a symmetry index of 0.8 mm. The symmetry index of the FR4 and WF sponge cakes with a puffy loaf was 6.4 mm and 17.5 mm, respectively, indicating that the height of the center of the WF sponge cake was the highest. The uniformity index, a measure of the lateral symmetry of the cake [44], ranged from −0.3 to 1.0 mm for FRP sponge cakes and was 0.4 mm for the WF sponge cake. Notably, the uniformity index of the FR3 sponge cake with a flat loaf was 0.0 mm. These results indicate that all sponge cakes have a laterally symmetrical structure due to their uniformity index being closer to 0 [44]. However, considering both symmetry and uniformity indexes, uniform volume growth of batters and stable structure maintenance during baking were achieved only for FR3, FR4, and WF, as shown in Figure 5.

These morphological characteristics of the FRP sponge cakes, compared to the WF sponge cake, may result from differences in the rheological behaviors of FRP according to particle size [14,15,16,20]. In this study, FRP is gently folded into the whole egg foam using a spatula to minimize foam collapse. As a result, the FRP may be present on the surfaces of the foam and at the interfaces between bubbles. During baking, RFP gelatinizes before the egg protein begins to coagulate, reinforcing the foam structure and transitioning the sol-like batter into the gel-like structure (cake). The continuous gel network suppresses excessive bubble expansion, preventing air leakage and bubble merging. This leads to even better expansion and a stable, sturdy cake structure with smaller, evenly distributed air cells [13,14,16]. This is supported by previous studies [14,16,20], which demonstrated that relatively symmetric morphology without loaf collapse, enhanced specific volume, and smaller, more homogenous air cells in gluten-free rice cakes were achieved by increased viscosities or G′ values (in the frequency sweep test) of rice flour-based batters. Therefore, the absence of loaf collapse, relatively smaller air cells, and balanced morphology of sponge cakes made with FR3 and FR4 may be attributed to the higher peak and final viscosities of their pastes, as well as their greater resistance to deformation, higher elastic character, and increased strength in their gels.

#### 2.2.2. Quality Characteristics

The baking loss, moisture content, specific volume, and firmness of FRP and WF sponge cakes are presented in Table 6. The baking loss of FRP sponge cakes ranged from 11.6 to 13.1% with no significant difference, consistent with the results of the previous studies [6,7,16] that reported no direct relationship between the particle size of rice flour and the weight loss of gluten-free rice breads and cakes. There was no significant difference in baking loss between FRP and WF sponge cakes.

The moisture content of FRP sponge cakes increased from 24.8 to 29.5% with decreasing particle size. The WF sponge cake had a similar moisture content to that of FR3 and FR4 sponge cakes, which did not experience loaf collapse. Previous studies [6,7,16] reported that an increase in the specific volume of gluten-free rice breads and cakes increased their surface areas, accelerating moisture evaporation. However, in this study, the moisture content of FRP sponge cakes does not appear to be directly associated with their specific volume. A possible explanation is that the loaf collapses in FR1 and FR2 sponge cakes (Figure 5) reducing their overall volumes, facilitating moisture leaching, and ultimately leading to moisture loss.

The specific volume of FRP sponge cakes ranged from 2.4 to 3.3 mL/g, with significant differences observed. An increasing trend in specific volume was noted with decreasing FRP particle size. This result is consistent with the reports of the previous studies [6,7,13,16] that demonstrated finer rice flour enhances the specific volume of gluten-free rice breads and cakes. Finer rice flour exhibits higher peak and final viscosities and greater resistance to deformation, contributing to gas retention in gluten-free breads and cakes [6,7,13,16]. Additionally, its gel has higher G′ and G″ values with a gel-like behavior—between liquid-like and solid-like, where G′ > G″—and develops a greater G′ value during cooling after heating, maintaining the expanded structure of the cakes with minimal collapse [6,13]. These findings are consistent with those for FR3 and FR4, as the FR3 and FR4 sponge cakes, without loaf collapse, had significantly higher specific volumes than the FR1 and FR2 sponge cakes. Meanwhile, the WF sponge cake exhibited a significantly higher specific volume than the FRP sponge cakes. This may be due to the presence of gluten, even though gluten development in WF is minimized during sponge cake preparation. The pasting viscosity (Figure 1) and G′ values within LVR (Figure 2), as well as G′ values in the frequency sweep test (Table 4) for WF paste and gel, were lower than those of FR3 and FR4. However, the final G′ value in the temperature sweep profile (Figure 3) and the C5 value in the Mixolab profile (Figure 4) exceeded those in FR3 and FR4, likely due to the presence of gluten. Additionally, even underdeveloped gluten contributes to the setting of batters that expand during baking [13].

The firmness of FRP sponge cakes significantly decreased from 6.1 to 2.9 N with decreasing FRP particle size, consistent with the results of the previous studies [13,16]. This result is due to an increase in specific volume and a decrease in air cell size of the FRP sponge cakes as the FRP particle size decreases [13,16]. Thus, the WF sponge cake, with the highest specific volume and smallest air cell size, exhibited significantly lower firmness (1.5 N) compared to the FRP sponge cakes (2.9–6.1 N). Meanwhile, as previously mentioned in Section 2.1.3, the finer FRF initiated gelatinization at a relatively lower temperature, but differences in gelatinization peak and completion temperatures, as well as gelatinization and melting enthalpies, were negligible among FRF fractions. Consequently, the quality attributes of FRF sponge cakes appear to be minimally influenced by the gelatinization and retrogradation properties of FRF based on its particle size.

Further comparing the findings of this study with bakery products made with other gluten-free flours, de la Hera et al. (2013) [21] demonstrated that using yellow and white maize flour with different particle sizes for gluten-free breadmaking increased the viscoelastic attributes of the dough with decreasing particle size. This resulted in enhanced specific volume and reduced firmness in yellow maize flour-based gluten-free bread, consistent with the findings observed in FRF fractions. Gadallah (2017) [27] reported that when rice flour was partially replaced with sorghum flour and germinated chickpea flour, the pasting viscosity of the mixtures and the specific volume of the cake decreased as the content of sorghum or germinated chickpea flour increased. This relationship between pasting viscosity and specific volume is similar to the results of this study, where an increase in the pasting viscosity and viscoelastic attributes of FRF led to an increase in the specific volume of the sponge cake. Curti et al. (2022) [28] demonstrated that finer sorghum flour produced cake batter with higher viscosity and smaller, more uniform bubbles. However, they reported no relationship between sorghum flour particle size and the quality attributes of gluten-free sponge cake, achieving acceptable cake qualities at a specific particle size distribution. Sung et al. (2020) [29] showed that increasing the addition of chia seed flour to rice flour-chia seed flour mixtures in gluten-free layer cake making increased batter viscosity and cake hardness but decreased the specific volume of the cake. Taromsari and Tarzi (2024) [23] demonstrated that batter viscosity decreased with decreasing coconut flour content or with increasing rice flour or xanthan gum at various mixing ratios of rice flour, coconut flour, and xanthan gum. However, the quality characteristics of gluten-free cakes were not consistent based on flour content; instead, an optimal mixing ratio was identified to achieve acceptable cake qualities. Overall, the finding that the pasting and viscoelastic properties of FRF are directly related to the quality of gluten-free sponge cake does not appear to be universally applicable to all other gluten-free flours.

The absence of wheat flour in gluten-free bakery products results in a lack of gluten, which provides essential protein and crucial functions, such as gas retention, viscoelastic dough development, and structure setting during baking [1]. Dhen et al. (2016) [26] demonstrated that the partial replacement of corn starch with soy flour showed no consistent cake batter viscosities based on particle size. However, the specific volume of sponge cakes decreased with decreasing particle size, partially aligning with the results of this study. Sahagún et al. (2018) [14] investigated the effects of adding plant (pea and rice) and animal (egg white and whey) protein on gluten-free layer cake qualities to fortify protein. Plant proteins decreased cake volume, whereas animal proteins increased it. Cake hardness increased with the addition of protein. This suggests that the gelatinization, pasting, viscoelastic behavior, and gelation properties of the flour mixture during baking are more important than the rheological properties of the batter. Ostermann-Porcel et al. (2020) [25] showed that adding okara flour reduced the batter viscosity of layer cakes as particle size increased, while the specific volume of the cake generally increased and hardness decreased. This result was partially consistent with that of this study. The discrepancy may be due to the characteristic of okara itself, which has lower solubility and only swells to a limited extent, failing to develop viscosity. Meanwhile, the deterioration of final qualities in gluten-free bakery products due to the lack of gluten can be improved by adding hydrocolloids [3]. These hydrocolloids increase batter viscosity, enhancing gas retention and preventing bubble coalescence. This increases the specific volume of bakery products, resulting in lower firmness. However, each hydrocolloid has a maximum effective concentration for achieving acceptable gluten-free bakery product qualities, as excessive use can degrade the quality. Xanthan gum, guar gum, sodium alginate, hydroxypropyl methylcellulose (HPMC), and carrageenan have been reported to positively affect the quality of gluten-free bakery products at concentrations ranging from 0.1% to 2.0% [3,22,23,24].

## 3. Conclusions

This study demonstrated the impact of FRP particle size on the quality characteristics of gluten-free butter sponge cake and verified the relationship between the pasting and rheological properties of FRP and the quality attributes of the cake. As the particle size of FRP decreased, its solubility, pasting viscosity, resistance to deformation, and viscoelastic gel-like behavior were enhanced. Additionally, the particle size of FRP directly influenced loaf development and stability, morphological balance, specific volume, and firmness of gluten-free sponge cakes. These attributes were improved with decreasing FRP particle size, correlating with trends in solubility, pasting viscosity, and viscoelastic parameters (in the strain, frequency, and temperature sweep tests). Overall, these findings suggest that the quality attributes of gluten-free rice cakes, particularly in sponge cakes where FRP is gently folded into the whole egg foam using a spatula, can be predicted by measuring rheological characteristics of the FRP paste and gel without analyzing the rheological behavior of the oily cake batter. The results and analysis methods in this study could serve as criteria for selecting flour sources to replace soft wheat flour in gluten-free sponge cakes. Moreover, this study recommends using rice flour that passes through at least an 80-mesh sieve (180 μm pore size) when making gluten-free rice sponge cakes. Furthermore, floury rice powder (FR3) with a particle size of 80–100 mesh seems to be the appropriate raw material for gluten-free sponge cake to minimize cutting loss in the production of decorated layer cakes. Future research should focus on exploring combinations of FRP and hydrocolloids to produce gluten-free sponge cakes that approximate the specific volume and firmness of wheat flour-based sponge cakes by investigating the rheological properties of FRP and hydrocolloid mixtures.

## 4. Materials and Methods

### 4.1. Materials

Floury rice grain (Baromi2 variety), cultivated in October 2023 and polished in May 2024, was obtained from Cheongwon Life Nonghyup Co., Ltd. (Cheongju, Republic of Korea). Soft wheat flour (WF) was purchased from Daehan Flour Mills (Incheon, Republic of Korea). Commercial products such as eggs, sucrose, butter, salt, and baking powder used for preparing butter sponge cake were purchased from a local market in Suwon, Republic of Korea. All chemicals and reagents used in this study were of ACS grade.

### 4.2. Preparation and Fractionation of Floury Rice Powder (FRP)

FRP was prepared by dry milling using a roll mill (DK104; Dongkwang Co., Hwaseong, Republic of Korea). Floury rice grain (1 kg, 11.3% moisture content) was initially milled at a roll gap of 5 mm. The coarse FRP was then milled again at a roll gap of 3 mm, followed by a final milling at a roll gap of 1 mm. The resultant FRP was sieved using a vibratory sieve shaker (AS 200 Basic; Retsch GmbH, Haan, Germany) equipped with standard sieves of 60 (pore size: 250 μm), 80 (pore size: 180 μm), and 100 mesh (pore size: 150 μm) (Chunggye Sieve Co., Gunpo, Republic of Korea). The sieve shaker was operated for 2 min at 25% amplitude with a 10 s pulse for each 200 g trial of FRP. This operation was repeated until at least 1 kg of each FRP fraction was achieved. The FRP fractions were classified into 60-mesh overs, 60–80 mesh, 80–100 mesh, and 100-mesh throughs, designated as FR1, FR2, FR3, and FR4, respectively. The FRF fractions were transferred into PET sample bottles, tightly sealed, and stored at 4 °C prior to use.

### 4.3. Characteristics of FRP Fractions

#### 4.3.1. Chemical Composition

Crude protein, crude fat, and crude ash contents of FRP fractions and WF were assayed using the Kjeldahl method (%N × 5.95 for FRP fractions and %N × 5.83 for WF), the Soxhlet method with diethyl ether, and the dry ashing method at 550 °C, respectively [45]. Carbohydrate contents were determined by subtracting the sum of crude protein, fat, and ash contents (on a dry weight basis or d.b) from 100. Total starch contents were measured using a total starch assay kit (Megazyme Ltd., Wicklow, Ireland) according to AACC Approved Methods 76–13 [45]. Total dietary fiber contents were analyzed at the Korea Food Research Institute (Wanju, Jeollanam, Republic of Korea) according to AOAC Official Method 991.43 [46].

#### 4.3.2. Swelling Power and Solubility

The FRP fraction or WF (0.5 g, d.b) was mixed with deionized water (DIW; 25 mL) in a 50 mL conical tube and heated in a boiling water bath set at 100 °C with vortexing at 1-min intervals for the first 5 min and subsequently at 5-min intervals. The resultant paste was cooled to 20 °C and centrifuged for 20 min at 2500× *g* and 25 °C. The supernatant was carefully transferred into a 100 mL volumetric flask and diluted to 100 mL by adding DIW. The precipitate was weighed, and the total carbohydrate content in the diluted supernatant was quantified using a sulfuric acid–phenol colorimetric method [47]. The swelling power and solubility were determined as follows [36]:

$$Swelling\;power\left(g/g\right)=\frac{Precipitate\;weight\left(g,w.b\right)}{Initial\;sample\;weight\left(g,d.b\right)\times \left(1-\frac{\%Solubility}{100}\right)}$$

$$Solubility\left(\%\right)=\frac{Total\;carbohydrate\;weight\left(g\right)\;in\;the\;diluted\;supernatnat\times 0.9}{Initial\;sample\;weight(g,d.b)}\times 100$$

where 0.9 is the ratio of the molar masses of anhydroglucose (162 g/mol) to glucose (180 g/mol).

#### 4.3.3. Differential Scanning Calorimetry (DSC)

The gelatinization and retrogradation properties of the FRP fractions and WF were examined using DSC (DSC 4000; PerkinElmer Inc., Waltham, MA, USA) [40]. The sample (5 mg, d.b) was directly weighed into a stainless-steel pan, and DIW was added to achieve a total weight of 20 mg. The pan was hermetically sealed and held for 18 h at 22 °C to allow for equilibrium moisture. Then, the pan was scanned at a heating rate of 5 °C/min from 30 to 150 °C to obtain the DSC thermogram. The scanned pan was stored at 4 °C for 1 week and rescanned under the same conditions. An empty pan was used as a reference.

#### 4.3.4. Rapid Visco Analyzer (RVA)

The pasting viscosity profiles and parameters of FRP fractions and WF were investigated using RVA (RVA 4800; PerkinElmer Co., Springfield, IL, USA) [48]. The sample (2.1 g, d.b) was directly weighed into an aluminum canister, and DIW was added to adjust the total weight of 28 g (total solid content: 7.5%). The mixture was manually stirred for 30 s with a spatula to ensure homogenous dispersion, followed by RVA testing. The RVA analysis was conducted under a constant rotation speed of 160 rpm using a plastic paddle according to the predetermined temperature profile: holding for 2 min at 50 °C, heating to 95 °C at a heating rate of 12 °C/min, holding for 2.5 min at 95 °C, cooling to 50 °C at a cooling rate of 12 °C/min, and holding for 2 min at 50 °C.

#### 4.3.5. Viscoelastic Measurement

The rheological characteristics of FRP fractions and WF were investigated using a dynamic rheometer (DHR1; TA Instruments, New Castle, DE, USA) equipped with a parallel Peltier plate-plate geometry system (40 mm diameter) [41]. The gap between the Peltier plate and geometry was 1000 μm, and a solvent trap module was used to inhibit moisture evaporation during testing. For the temperature sweep test, the sample was mixed with DIW in a 2 mL Eppendorf tube at a total solid content of 15%, vortexed, and hydrated for 30 min at 22 °C. An aliquot of the suspension was placed on the Peltier plate and conditioned for 2 min at 40 °C. The suspension was then oscillated at 1 Hz and 1% strain (within the linear viscoelastic range) across the predetermined temperature profile: heating from 40 to 95 °C at a heating rate of 2 °C/min, holding at 95 °C for 5 min, and cooling from 95 to 5 °C at a cooling rate of 2 °C/min. For the strain sweep test, the sample was mixed with DIW in a 50 mL conical tube at a total solid content of 7.5%. The suspension was heated for 30 min in a boiling water bath set at 100 °C with 5-min intervals and cooled in a cold-water bath (approximately 18 °C). The resultant gel was oscillated at 1 Hz across a strain range of 0.001–100%, following conditioning for 5 min at 25 °C. For the frequency sweep test, the fresh gel, identical to that prepared in the strain sweep test, was oscillated at 1% strain across an angular frequency range of 0.1–100 rad/s, following conditioning for 5 min at 25 °C.

#### 4.3.6. Thermo-Mechanical Measurement

The thermos-mechanical properties of FRP fractions and WF were investigated using Mixolab (Chopin, Tripette et Renaud, Paris, France) [31]. Each sample was blended with DIW in a Mixolab bowl to obtain 90 g of dough at 70% and 49.8% water absorption for the FRP fractions and WF, respectively. The changes in torque generated by continuous mixing (120 rpm) were tracked across the programmed temperature profile: holding for 8 min at 30 °C, heating to 90 °C at a heating rate of 4 °C/min, holding for 7 min at 90 °C, and cooling to 50 °C at a cooling rate of 4 °C/min, and holding for 10 min at 50 °C.

### 4.4. Preparation and Characteristics of Butter Sponge Cakes

#### 4.4.1. Preparation of Butter Sponge Cakes

Butter sponge cake was prepared using a whole-egg foam method. Whole egg (450 g), sucrose (360 g), and salt (6 g) were placed in a mixing bowl and double-boiled for 5 min using a water bath set at 45 °C. The mixture was then whipped for 15 min at full speed (speed 3) using a Spar mixer (SP-800; SPAR Food Machinery MFG Co., Ltd., Taichung, Taiwan) equipped with a wire whisk to achieve maximum volume. Afterwards, the pre-sieved mixture of FRP fractions or WF (300 g) and baking powder (1.5 g) was incorporated into the whole egg foam, followed by adding the butter (90 g) melted at 50–60 °C, and manually stirred using a spatula without knocking out air. The specific gravity of the resultant batter was between 0.5 and 0.6. Equal amounts of the batter were transferred into four 8-inch-diameter round pans and baked for 30 min in a conventional oven set at 180 °C on the top and 160 °C on the bottom. The baked butter sponge cakes were removed from the pans and cooled for 1 h at 22 °C prior to analysis.

#### 4.4.2. Appearance Characteristic

Each butter sponge cake was cut through the center, and the heights at five distinct positions (A–E) in the cross-section were measured using a layer cake measuring template. The volume, symmetry, and uniformity indexes of the cakes were calculated according to AACC Approved Method 10–91 [45]. Cross-section images were taken using a scanner (SLM2074, Samsung, Suwon, Republic of Korea), and the crumbs were observed using a stereomicroscope (SMZ745T, Nikon Co., Tokyo, Japan) at 6.7× magnification under two reflected LED illuminations (3 W each).

#### 4.4.3. Moisture Content, Baking Loss, and Specific Volume

The moisture content of the cake crumb was measured using an infrared moisture analyzer (MA37; Sartorius AG, Göttingen, Germany) at 105 °C. Baking loss and specific volume were determined according to the procedures outlined in AACC Approved Method 10–90.01 and 72–10, respectively [45].

#### 4.4.4. Firmness

The cake was cut into cubes (20 mm × 20 mm × 20 mm). Each specimen was compressed to 50% deformation at a crosshead speed of 5 mm/s using a texture analyzer (Z0.5 TS; Zwick Roell AG, Ulm, Baden-Württemberg, Germany) equipped with a P/100 probe (100 mm in diameter). Firmness was defined as the peak force obtained from the force-time profile.

### 4.5. Statistical Analysis

The preparation of FRP fractions were repeated until at least 1 kg of each fraction was obtained, and butter sponge cakes were prepared three times. All characteristic measurements were repeated at least three times per treatment. All data were analyzed using one-way analysis of variance (ANOVA) and expressed as mean ± standard deviation. Significant differences in mean values were identified using Duncan’s multiple range test at *p* < 0.05. All statistical computations and analyses were conducted using SPSS (version 23.0; IBM-SPSS Inc., Chicago, IL, USA).

## Figures and Tables

**Table 1:** Chemical compositions * of soft wheat flour and floury rice powder (FRP) fractionated by particle size distribution.

FRP Fraction ^1^	Crude Protein (%, d.b)	Crude Lipid (%, d.b)	Crude Ash (%, d.b)	Carbohydrate (%, d.b)	Total Starch (%, d.b)	Total Dietary Fiber (%, d.b)
FR1	8.1 ± 0.0 ^a^	2.2 ± 0.1 ^a^	0.5 ± 0.0 ^a^	89.2 ± 0.1 ^d^	73.4 ± 1.0 ^b^	1.7 ± 0.3 ^a^
FR2	7.6 ± 0.6 ^a^	2.0 ± 0.0 ^b^	0.5 ± 0.0 ^a^	89.9 ± 0.1 ^c^	76.1 ± 2.0 ^a^	1.8 ± 0.2 ^a^
FR3	7.6 ± 0.6 ^a^	1.6 ± 0.1 ^c^	0.5 ± 0.0 ^a^	90.3 ± 0.3 ^c^	77.6 ± 1.2 ^a^	1.8 ± 0.0 ^a^
FR4	5.8 ± 0.6 ^b^	1.6 ± 0.0 ^c^	0.5 ± 0.0 ^a^	92.1 ± 0.1 ^a^	77.6 ± 1.1 ^a^	1.9 ± 0.1 ^a^
WF ^2^	7.3 ± 0.0 ^a^	0.9 ± 0.1 ^d^	0.3 ± 0.0 ^b^	91.5 ± 0.1 ^b^	71.6 ± 1.4 ^c^	2.1 ± 0.3 ^a^

* Mean values of three replicate measurements; values sharing the same lowercase letters are not significantly different at *p* < 0.05. ^1^ FR1, FR2, FR3, and FR4 represent FRPs fractionated to greater than 60 mesh, between 60 and 80 mesh, between 80 and 100 mesh, and less than 100 mesh, respectively. ^2^ WF indicates soft wheat flour as a control.

**Table 2:** Swelling power and solubility * of soft wheat flour and floury rice powder (FRP) fractionated by particle size distribution.

FRP Fraction ^1^	Swelling Power (g/g)	Solubility (%, d.b)
FR1	22.2 ± 0.4 ^a^	12.3 ± 0.0 ^d^
FR2	22.2 ± 0.2 ^a^	13.8 ± 0.7 ^c^
FR3	22.5 ± 0.0 ^a^	13.5 ± 0.1 ^c^
FR4	22.3 ± 0.6 ^a^	15.1 ± 0.7 ^b^
WF ^2^	16.4 ± 0.4 ^b^	20.0 ± 1.3 ^a^

* Mean values of three replicate measurements; values sharing the same lowercase letters are not significantly different at *p* < 0.05. ^1^ FR1, FR2, FR3, and FR4 represent FRPs fractionated to greater than 60 mesh, between 60 and 80 mesh, between 80 and 100 mesh, and less than 100 mesh, respectively. ^2^ WF indicates soft wheat flour as a control.

**Table 3:** Gelatinization property and melting enthalpy * of soft wheat flour and floury rice powder (FRP) fractionated by particle size distribution.

FRP Fraction ^1^	Gelatinization Temperature (°C)			Gelatinization Enthalpy (J/g)	Melting Enthalpy (J/g)
	Onset	Peak	Completion		
FR1	57.7 ± 0.9 ^a^	68.0 ± 0.3 ^a^	74.3 ± 0.1 ^ab^	7.2 ± 1.7 ^a^	1.4 ± 0.1 ^a^
FR2	56.5 ± 0.2 ^ab^	67.8 ± 0.0 ^ab^	74.7 ± 0.2 ^a^	9.1 ± 0.2 ^a^	1.2 ± 0.1 ^a^
FR3	56.4 ± 0.1 ^ab^	67.4 ± 0.0 ^b^	73.8 ± 0.3 ^ab^	8.8 ± 1.8 ^a^	1.2 ± 0.0 ^a^
FR4	55.4 ± 0.1 ^b^	67.7 ± 0.2 ^ab^	74.1 ± 0.2 ^ab^	8.6 ± 1.8 ^a^	1.4 ± 0.3 ^a^
WF ^2^	57.9 ± 0.1 ^a^	64.6 ± 0.6 ^c^	70.7 ± 0.2 ^b^	3.2 ± 0.0 ^b^	0.5 ± 0.0 ^b^

* Mean values of three replicate measurements; values sharing the same lowercase letters are not significantly different at *p* < 0.05. ^1^ FR1, FR2, FR3, and FR4 represent FRPs fractionated to greater than 60 mesh, between 60 and 80 mesh, between 80 and 100 mesh, and less than 100 mesh, respectively. ^2^ WF indicates soft wheat flour as a control.

**Table 4:** Frequency sweep parameters * of soft wheat flour and floury rice powder (FRP) fractionated by particle size distribution.

FRP Fraction ^1^	Storage Modulus (G′) (Pa)	Loss Modulus (G″) (Pa)	tan δ	Complex Viscosity (η*) (Pa·s)
FR1	47.1 ± 7.1 ^ab^	9.3 ± 1.7 ^ab^	0.2 ± 0.0 ^a^	7.6 ± 1.2 ^ab^
FR2	53.6 ± 3.0 ^ab^	10.7 ± 0.5 ^ab^	0.2 ± 0.0 ^a^	8.7 ± 0.5 ^ab^
FR3	62.7 ± 5.2 ^a^	11.3 ± 0.9 ^a^	0.2 ± 0.0 ^a^	10.1 ± 0.8 ^a^
FR4	63.0 ± 9.1 ^a^	12.6 ± 2.4 ^a^	0.2 ± 0.0 ^a^	10.2 ± 1.5 ^a^
WF ^2^	53.0 ± 5.9 ^ab^	12.4 ± 1.9 ^a^	0.2 ± 0.1 ^a^	8.6 ± 2.5 ^ab^

* Mean values of three replicate measurements; values sharing the same lowercase letters are not significantly different at *p* < 0.05. ^1^ FR1, FR2, FR3, and FR4 represent FRPs fractionated to greater than 60 mesh, between 60 and 80 mesh, between 80 and 100 mesh, and less than 100 mesh, respectively. ^2^ WF indicates soft wheat flour as a control.

**Table 5:** Volume, symmetry, and uniformity indexes * of butter sponge cakes prepared using soft wheat flour and floury rice powder (FRP) fractionated by particle size distribution.

FRP Fraction ^1^	Volume Index (mm)	Symmetry Index (mm)	Uniformity Index (mm)
FR1	64.7 ± 7.9 ^e^	−8.1 ± 0.8 ^d^	−0.3 ± 0.4 ^b^
FR2	88.8 ± 2.3 ^d^	−10.0 ± 2.1 ^d^	−0.2 ± 0.3 ^b^
FR3	126.9 ± 4.9 ^b^	0.8 ± 0.0 ^c^	0.0 ± 0.1 ^b^
FR4	116.7 ± 3.2 ^c^	6.4 ± 2.7 ^b^	1.0 ± 0.3 ^a^
WF ^2^	185.4 ± 4.8 ^a^	17.5 ± 7.9 ^a^	0.4 ± 0.3 ^ab^

* Mean values of three replicate measurements; values sharing the same lowercase letters are not significantly different at *p* < 0.05. ^1^ FR1, FR2, FR3, and FR4 represent FRPs fractionated to greater than 60 mesh, between 60 and 80 mesh, between 80 and 100 mesh, and less than 100 mesh, respectively. ^2^ WF indicates soft wheat flour as a control.

**Table 6:** Baking loss, moisture content, specific volume, and firmness * of soft wheat flour and floury rice powder (FRP) fractionated by particle size distribution.

FRP Fraction ^1^	Baking Loss (%)	Moisture Content (%)	Specific Volume (mL/g)	Firmness (N)
FR1	13.1 ± 0.1 ^a^	24.8 ± 1.0 ^b^	2.6 ± 0.0 ^c^	6.1 ± 0.7 ^a^
FR2	13.0 ± 0.4 ^a^	26.7 ± 1.9 ^ab^	2.4 ± 0.2 ^c^	4.2 ± 0.5 ^b^
FR3	11.6 ± 0.8 ^a^	28.5 ± 0.9 ^a^	3.3 ± 0.3 ^b^	3.3 ± 0.4 ^c^
FR4	13.0 ± 0.8 ^a^	29.5 ± 0.7 ^a^	2.9 ± 0.2 ^b^	2.9 ± 0.3 ^c^
WF ^2^	12.8 ± 1.7 ^a^	29.8 ± 0.7 ^a^	4.2 ± 0.2 ^a^	1.5 ± 0.6 ^d^

* Mean values of three replicate measurements; values sharing the same lowercase letters are not significantly different at *p* < 0.05. ^1^ FR1, FR2, FR3, and FR4 represent FRPs fractionated to greater than 60 mesh, between 60 and 80 mesh, between 80 and 100 mesh, and less than 100 mesh, respectively. ^2^ WF indicates soft wheat flour as a control.

## Data Availability

The data presented in this study are available upon request from the corresponding author.

## Supplementary Materials

The following supporting information can be downloaded at: https://www.mdpi.com/article/10.3390/gels11100789/s1, Figure S1: DSC thermograms of soft wheat flour (WF) and floury rice powder (FR1–FR4) fractionated by particle size distribution; Table S1: RVA parameters of soft wheat flour (WF) and floury rice powder (FR1–FR4) fractionated by particle size distribution; Figure S2: Changes in strain sweep parameters (G′ and G″) of soft wheat flour (WF) and floury rice powder (FR1–FR4) fractionated by particle size distribution; Figure S3: Frequency sweep profiles of soft wheat flour (WF) and floury rice powder (FR1–FR4) fractionated by particle size distribution; Figure S4: Changes in temperature sweep parameters (G′ and G″) of soft wheat flour (WF) and floury rice powder (FR1–FR4) fractionated by particle size distribution; Table S2: Mixolab parameters of soft wheat flour (WF) and floury rice powder (FR1–FR4) fractionated by particle size distribution.
