# Supplementary material for: Physicochemical and Rheological Properties of Floury Rice Powder with Different Particle Sizes: Effects on Gluten-Free Sponge Cake Qualities
Source: Gels. 2025 Oct 1;11(10):789. doi: 10.3390/gels11100789 (PMC12562764; doi:10.3390/gels11100789)
Supplement: Supplementary file 1 [file gels-11-00789-s001.zip › gels-3895773-supplementary.pdf]

Physicochemical and Rheological Properties of Floury Rice Powder with Different Particle Sizes: Effects on Gluten-Free Sponge Cake Qualities

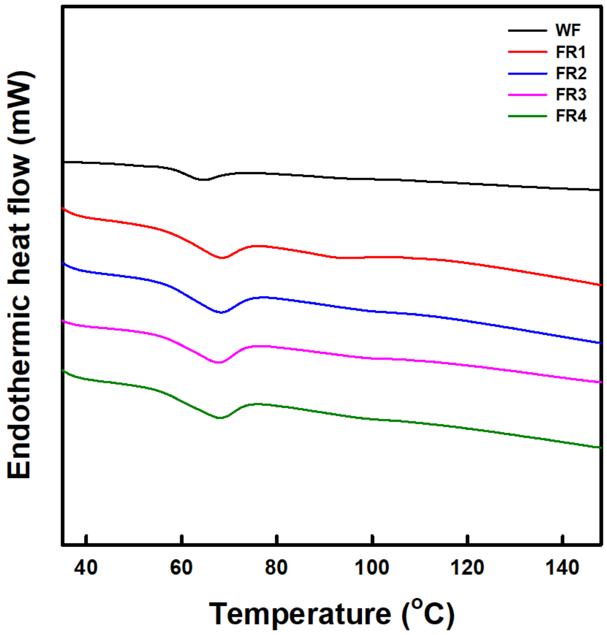

Figure S1. DSC thermograms of soft wheat flour (WF) and floury rice powder (FR1-FR4) fractionated by particle size distribution.

Table S1. RVA parameters of soft wheat flour (WF) and floury rice powder (FR1-FR4) fractionated by particle size distribution

FRP fraction[1]	Viscosity (mPa·s)				
	Peak	Trough	Breakdown	Final	Setback
FR1	929.0±7.7[d]	796.0±8.9[d]	133.0±2.0[c]	1,704.0±8.9[d]	908.0±4.6[d]
FR2	1,207.7±8.5[c]	985.3±2.8[c]	222.4±4.0[b]	2,167.3±7.5[c]	1,182.0±3.9[b]
FR3	1,312.3±6.8[b]	1,093.7±1.7[b]	218.6±5.5[b]	2,236.0±6.4[b]	1,142.3±5.0[c]
FR4	1,366.7±3.9[a]	1,102.0±3.9[a]	264.7±2.5[a]	2,342.0±3.0[a]	1,240.0±9.5[a]
WF[2]	573.0±5.4[e]	451.7±8.6[e]	121.3±3.2[d]	983.7±2.1[e]	532.0±1.5[e]

* Mean values of three replicate measurements; values sharing the same lowercase letters are not significantly different at $p<0.05$. [1] FR1, FR2, FR3, and FR4 represent FRPs fractionated to greater than 60 mesh, between 60 and 80 mesh, between 80 and 100 mesh, and less than 100 mesh, respectively. [2] WF indicates soft wheat flour as a control.

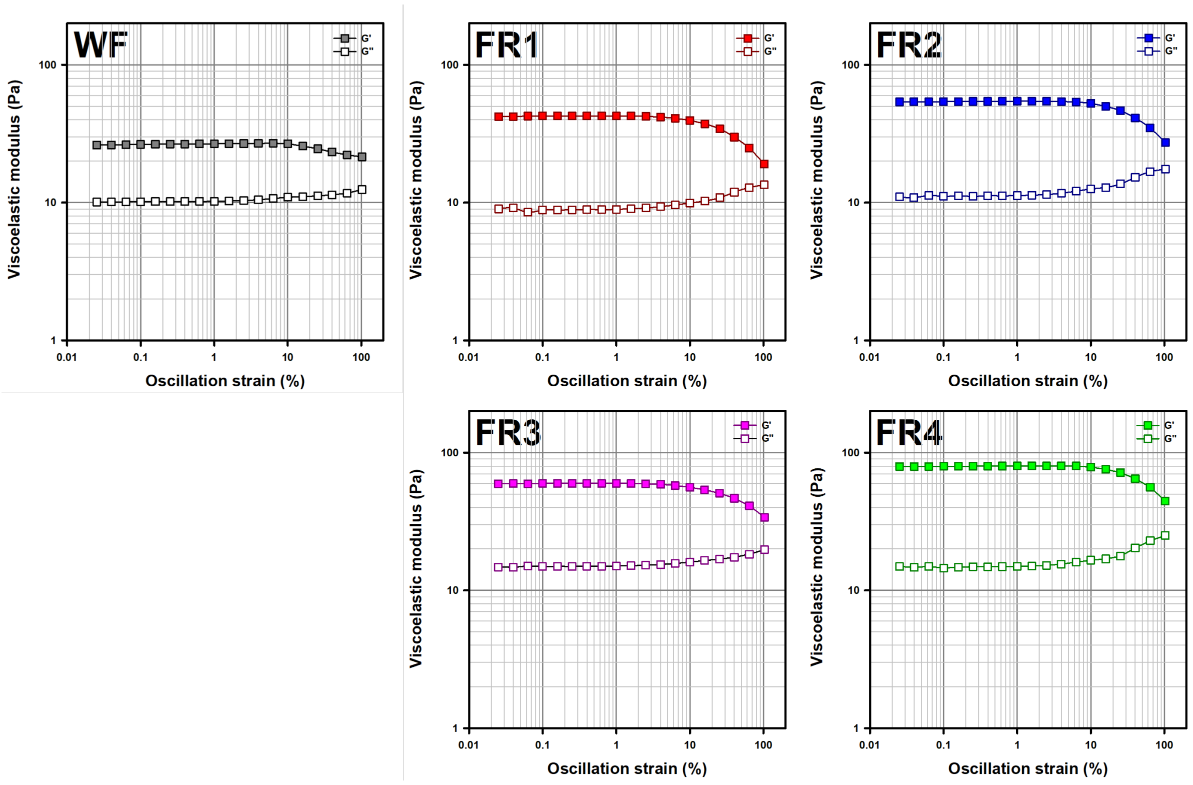

Figure S2. Changes in strain sweep parameters (G' and G") of soft wheat flour (WF) and floury rice powder (FR1-FR4) fractionated by particle size distribution.

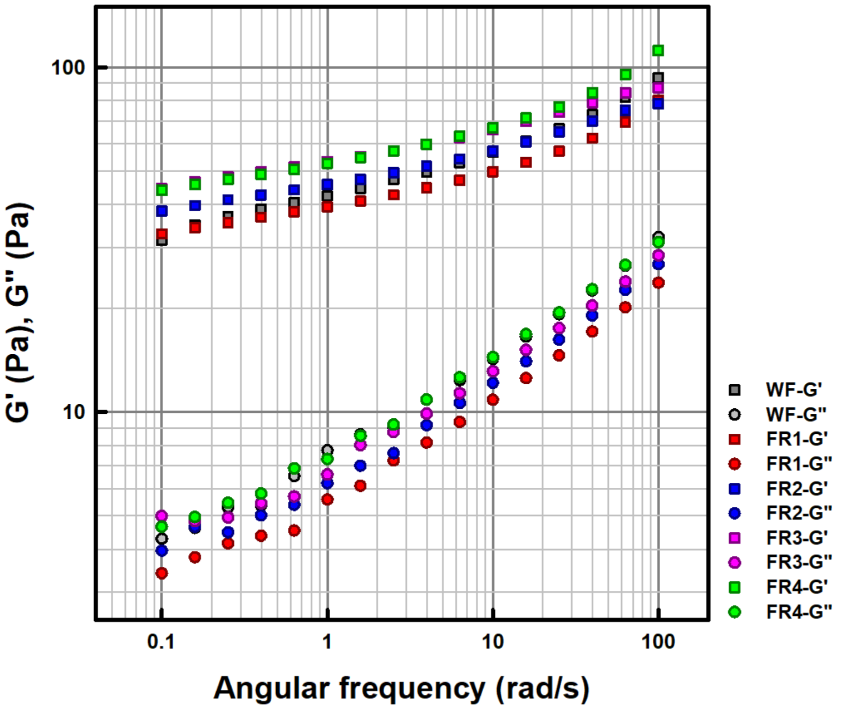

Figure S3. Frequency sweep profiles of soft wheat flour (WF) and floury rice powder (FR1-FR4) fractionated by particle size distribution.

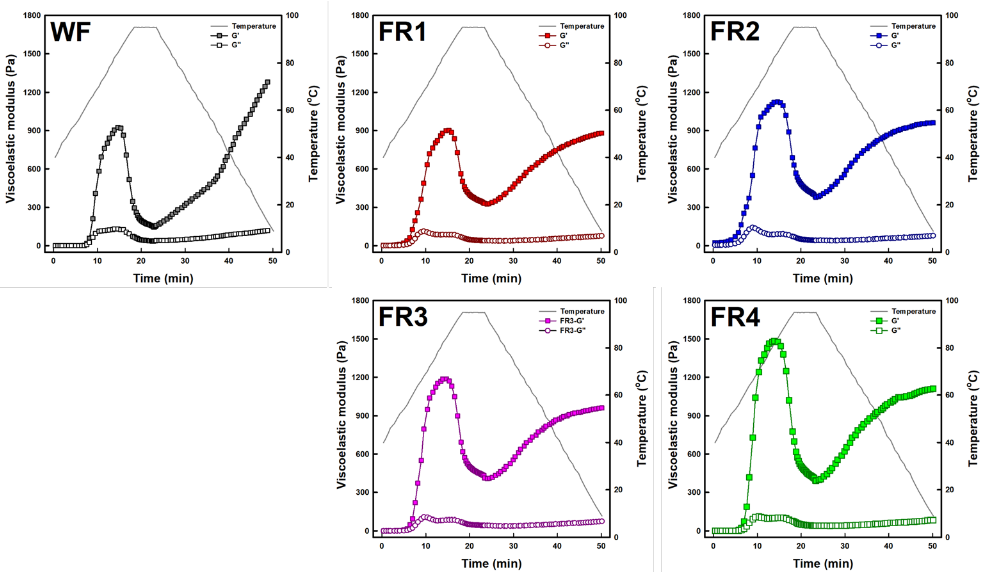

Figure S4. Changes in temperature sweep parameters (G' and G") of soft wheat flour (WF) and floury rice powder (FR1-FR4) fractionated by particle size distribution.

Table S2. Mixolab parameters of soft wheat flour (WF) and floury rice powder (FR1-FR4) fractionated by particle size distribution

FRP fraction[1]	Water absorption (%)	Dough development (min)	Stability (min)
FR1	70.0	8.39±0.04[a]	1.32±0.02[a]
FR2	70.0	0.51±0.01[c]	0.68±0.09[b]
FR3	70.0	0.52±0.00[c]	0.24±0.01[c]
FR4	70.0	0.60±0.14[c]	0.27±0.16[c]
WF[2]	49.8	1.30±0.09[b]	3.83±0.52[b]

FRP fraction[1]	C1 (N·m)	C2 (N·m)	C3 (N·m)	C4 (N·m)	C5 (N·m)
FR1	0.36±0.00[d]	0.10±0.00[e]	2.08±0.02[c]	1.44±0.01[c]	2.31±0.02[bc]
FR2	0.62±0.02[c]	0.13±0.00[d]	2.17±0.00[b]	1.50±0.03[b]	2.38±0.02[b]
FR3	1.06±0.04[b]	0.22±0.00[c]	2.08±0.00[c]	1.48±0.00[b]	2.34±0.02[bc]
FR4	1.46±0.11[a]	0.33±0.02[b]	2.08±0.06[c]	1.48±0.01[b]	2.32±0.02[bc]
WF[2]	1.08±0.01[b]	0.43±0.01[a]	2.27±0.00[a]	2.04±0.04[a]	3.91±0.07[a]

FRP fraction[1]	C1-C2 (N·m)	C3-C4 (N·m)	C5-C4 (N·m)	Slope-α	Slope-β	Slope-γ
FR1	0.16±0.00[e]	0.67±0.03[a]	0.88±0.01[b]	-0.04±0.00[a]	1.11±0.05[a]	-0.06±0.01[a]
FR2	0.49±0.02[d]	0.64±0.01[a]	0.87±0.00[b]	-0.05±0.01[ab]	0.79±0.42[a]	-0.06±0.02[a]
FR3	0.84±0.04[b]	0.60±0.00[a]	0.87±0.02[b]	-0.06±0.01[bc]	1.01±0.11[a]	-0.05±0.01[a]
FR4	1.13±0.14[a]	0.60±0.06[a]	0.84±0.01[b]	-0.07±0.01[c]	0.77±0.08[a]	-0.05±0.00[a]
WF[2]	0.65±0.00[c]	0.22±0.03[b]	1.87±0.03[a]	-0.07±0.00[a]	0.29±0.00[a]	-0.02±0.01[a]

* Mean values of three replicate measurements; values sharing the same lowercase letters are not significantly different at $p<0.05$. [1] FR1, FR2, FR3, and FR4 represent FRPs fractionated to greater than 60 mesh, between 60 and 80 mesh, between 80 and 100 mesh, and less than 100 mesh, respectively. [2] WF indicates soft wheat flour as a control.
